# Enriching IoT Modules with Edge AI Functionality to Detect Water Misuse Events in a Decentralized Manner

**DOI:** 10.3390/s22134874

**Published:** 2022-06-28

**Authors:** Dimitrios Loukatos, Kalliopi-Agryri Lygkoura, Chrysanthos Maraveas, Konstantinos G. Arvanitis

**Affiliations:** Department of Natural Resources Management and Agricultural Engineering, Agricultural University of Athens, 75 Iera Odos Str., Botanikos, 11855 Athens, Greece; stud616018@aua.gr (K.-A.L.); maraveas@aua.gr (C.M.); karvan@aua.gr (K.G.A.)

**Keywords:** water resource preservation, Internet of Things, Edge Computing, Machine Learning, Edge AI, Smart Sensing, Precision Agriculture, Arduino, Raspberry, Edge Impulse

## Abstract

The digital transformation of agriculture is a promising necessity for tackling the increasing nutritional needs of the population on Earth and the degradation of natural resources. Focusing on the “hot” area of natural resource preservation, the recent appearance of more efficient and cheaper microcontrollers, the advances in low-power and long-range radios, and the availability of accompanying software tools are exploited in order to monitor water consumption and to detect and report misuse events, with reduced power and network bandwidth requirements. Quite often, large quantities of water are wasted for a variety of reasons; from broken irrigation pipes to people’s negligence. To tackle this problem, the necessary design and implementation details are highlighted for an experimental water usage reporting system that exhibits Edge Artificial Intelligence (Edge AI) functionality. By combining modern technologies, such as Internet of Things (IoT), Edge Computing (EC) and Machine Learning (ML), the deployment of a compact automated detection mechanism can be easier than before, while the information that has to travel from the edges of the network to the cloud and thus the corresponding energy footprint are drastically reduced. In parallel, characteristic implementation challenges are discussed, and a first set of corresponding evaluation results is presented.

## 1. Introduction

The degradation of natural resources in quality and quantity has a direct impact on the global food production numbers. According to FAO [1], the agricultural sector should increase its productivity by 60 per cent to counterbalance the depletion of natural resources and the population growth on Earth. The utilization of innovative technologies seems to be a key factor for addressing these issues. In this regard, toward a successful digital transformation of agriculture, it is promising that the rapid development of the electronics industry has managed to increase the production numbers and the quality of several components, such as microcontroller units (MCUs), single board computers, sensors, and radio transceivers, at very affordable cost levels. More specifically, the recently appeared new generation of microcontrollers, apart from orchestrating typical sensing and acting tasks, can support composite operations at reduced execution times, as they have faster and more efficient processors and larger memory. In parallel, the advances in radio technology deliver low-power modules capable of long-range communication at reduced energy levels. These high-end components are not only widely available but are also accompanied by very fluent documentation and software tools that facilitate their programming, leading to improved implementations. These characteristics can lead to a more efficient approach regarding serious problems, such as the preservation of natural resources. Nevertheless, any fusion of software and hardware elements has first to address potential implementation bottlenecks, prior to the delivery of any effective solution.

Indeed, as the world will be populated by billions of connected devices [2] of limited resources, interacting with the surrounding environment and users, the bottleneck will be the increased amount of data traffic that could congest the network and generate several latency, reliability and privacy problems [3,4]. The deployment of enhanced processing features on Internet of Things (IoT) devices, for example Machine Learning (ML), reduces the network congestion by allowing computations to be performed close to the data sources, and thus it preserves privacy in uploading data, and reduces power consumption for wireless transmission to gateways or cloud servers [4]. In this regard, one of the options is to run the intelligent algorithms locally on the end devices (e.g., on the sensor nodes hardware). If the tasks are performed by smaller devices, less power will be required to keep them running and more flexible energy management will be applied, compared with the typical central system case. Small devices can operate on batteries for months or even for years, while a diverse set of energy harvesting options is offered for elongated operation duration. Thankfully, the recent technological advances delivered end devices with improved hardware characteristics (i.e., processing capabilities and memory size), thus making it possible for these devices to execute machine learning algorithms in an efficient and cost-effective manner. Not only do the microcontrollers become better performing, but the application of machine learning techniques on them, such as the artificial neural networks (ANNs), have also become more efficient, due to the improvement of the corresponding software platforms and tools.

In greater detail, the execution/utilization phase of an ANN requires less computational power than its training phase. In fact, during the training, a large amount of data is used to calculate the weights and biases of the network, and thus a quite powerful machine is needed. Once the learning has been completed and the network has been trained, the model can be used for inference actions with lower computational requirements [4]. Consequently, the AI algorithms can more likely be run on devices with less resources, as microcontrollers, allowing local data processing. Nevertheless, as the trained models may still remain comparatively heavy for the in situ MCUs, tools such as TensorFlow Lite [5], in the context of TinyML [6], make possible the creation of trimmed-down versions that can be fit safely in the improved generation of MCUs, but still of limited computational and memory capacity.

Finally, the improved transmission range characteristics of the low power wide area network (LPWAN) technologies, such as LoRa, perfectly fit to the reduced network traffic profiles [7]. The balanced utilization of the discussed technological innovations can deliver applications that can be very helpful for solving real-world problems, e.g., the preservation of water resources.

Water is one of the most critical resources on the Earth as, apart from humans, both plants and animals depend on it, while many processes from irrigation to washing or food preparation, cannot be accomplished without it. Despite its necessity, large amounts of water are being wasted due to a variety of reasons, from water pipe or valve failures to human inattention. It is noteworthy that according to the World Bank [8], the non-revenue water (NRW) level in developing countries ranges from 40% to 50% of the water pumped into the distribution systems. Furthermore, 80 per cent of wastewater in the world flows back into the ecosystem without being treated or reused, and 70 per cent of the world’s natural wetland extent has been lost [9]. Sustainable Development Goal 6 (SDG 6) [9] on water and sanitation, adopted by United Nations (UN) Member States as part of the 2030 Agenda for Sustainable Development [10], highlights in practice the importance of the proper water resource management, from both quantitative and qualitative perspective. As agriculture remains the largest consumer of water globally, the significance of water for keeping the food produce to satisfactory levels is crucial.

Targeted at the preservation of water resources with emphasis on their impact on agriculture, in this work, the pilot implementation of a smart water usage alerting system is presented. The whole approach exploits the findings of the approach described in [11] toward the delivery of a more compact and efficient solution with artificial intelligence (AI) capabilities. The latter task is addressed by utilizing recently-appeared, cost effective but powerful microcontroller boards and software, for supporting the in situ machine learning operations, and a low-power and long-range radio network technology based on the LoRa protocol. The combination of these elements results in reduced power consumption and in less network traffic and processing load for the central entities of the network, as the water usage classification decisions are taken locally, at the edges of the network, and only notification messages have to travel toward the end user. Response times are also reduced, while privacy is better preserved. The water usage episodes that the smart system had been trained to intercept were of comparatively short duration, but the methods being used and the accuracy being achieved make the proposed arrangements, only with minor modifications, to be applicable for supporting a wide variety of water preservation/misuse detection scenarios.

Apart from this introductory section, in order to better highlight the main objectives of this research, the rest of this paper is organized as follows: Section 2 highlights the motives and the challenges behind this work and the design directions being necessary. Section 3 provides interesting implementation details. Section 4 is dedicated to evaluation results and discussion. Finally, Section 5 contains important concluding remarks.

## 2. Background and Design Overview

### 2.1. Motives and Challenges for Agriculture

Internet of things (IoT) is an emerging technology that includes devices connected to the Internet equipped with sensors, transducers, radio transceivers, and actuators comprising a functioning of the whole that gathers, interchanges and responds to information [12]. In this regard, the IoT makes agricultural automation more efficient, and thus fosters production [13]. Recent works emphasize the contribution of the IoT technologies in critical agricultural operations [14,15], including precision farming, livestock, and greenhouses, with the irrigation and water management activities to be of among the open issues of growing interest [16].

Machine Learning (ML) is a very welcome companion for any IoT solution and provides multiple solutions to problems that were among the most difficult to be tacked without, some years ago. The exploitation of the ML potential by agriculture is a necessity that follows several directions [17], even beyond Agriculture 4.0 [18]. The most significant advantage of machine learning techniques is that they can provide generally applicable solutions, with minor human intervention and in a way that does not require meticulous a priori knowledge of the idiosyncracies of the system the solution is being tailored for. This makes satisfactorily-working solutions to be generated easily and quickly by people with less expertise in a specific area. Apparently, the role of the “experts” of the sector cannot be overlooked, but their involvement into the whole process remains consulting and supervising, as they do not have to inject “magic” threshold values into conventional and difficult to maintain blocks of code.

The Edge Computing (EC) is a newcomer to the equation of tackling modern problems more efficiently using IoT and ML. Indeed, a traditional IoT solution (a few years ago) typically required a large amount of real-time sensor data to be destined to a central computer entity in the cloud which in its turn had to process this increased amount of data, to take the necessary decisions and probably had to deliver the corresponding responses back to the appropriate nodes. This organization had to tackle high communication and processing loads, while any potential failure of the central entity would result in total system collapse. Furthermore, data privacy concerns were also very reasonable, as third-party communication, storage and/or decision entities had to get involved in the whole process. On the contrary, by increasing the intelligence at the edges of the network (i.e., on or nearby the sensor nodes), decisions and any potential action are addressed locally, in a faster, cheaper and more private way, thus leaving considerably less (or none at all) work for the central entity [4,19]. Typically, only sporadic metadata information updates are necessary toward the central entity, mainly for supervision purposes.

The enrichment of IoT with Edge Computing and Machine Learning functionality is often referred as Edge Artificial Intelligence (Edge AI) and tries to exploit the advantages of these technologies, for serving a wide set of applications in a better manner, with the agricultural sector not to be an exception [20]. In this regard, the approach being presented is trying to highlight how these elements of innovation can be combined to ease the intense problem of water resource waste.

Demographics continue changing and unsustainable economic practices are affecting the quantity and quality of the water being available, thus making it an increasingly scarce and expensive resource [9]. Inevitably, water is at the core of sustainable development and is closely linked to poverty reduction and climate change. As agriculture remains the largest consumer of water globally and irrigation is responsible for 70% of its use worldwide, water is the most valuable resource for keeping the quality and the quantity of plant and animal production to satisfactory levels. The way water is utilized for both urban and rural use directly impacts its future availability and thus, emphasis must be placed on water management and irrigation efficiency and make sure clean water can be provided for all people.

Apart from the more conventional bare IoT solutions for water resource management and utilization, mainly with focus on agriculture, there is a growing interest for the exploitation of ML in order to achieve better results [21,22,23,24]. The fusion with Edge AI functionality has yet a lot to offer. The potential exploitation of modern microcontrollers for water usage related applications with embedded ML functionality has already started delivering interesting outcomes [25], in neighboring scientific areas, with the selection of devices and functions for communication between sensor appliances to remain a key challenge [26] for success.

On the other hand, recent studies show that farmers are still facing concerns for adopting the IoT technologies in their everyday activities. This skepticism is attributed to a variety of reasons, from privacy concerns due the cloud-based nature of many solutions to fears for job cuts and for high purchase and maintenance costs [27,28], while it is really hard to find experts having the necessary set of talents at a satisfactory degree and being available for fluent cooperation, at the same time.

Furthermore, while the machine learning methods seem to provide accurate and less expensive solutions [23] for water misuse detection events such as leaks, there is enough room for further improvements. Indeed, due to the very recent character of the innovative hardware and software components supporting in situ (i.e., on-device node) machine learning techniques, in the agricultural sector for water utilization report/classification purposes, few works combine these assets toward the delivery of a cost effective and efficient solution with Edge AI characteristics. There are research contributions that exploit IoT infrastructures for water monitoring purposes, but without incorporating AI functionality [29] or there are contributions that exploit machine learning methods that either require central processing of the data being collected [30,31] or that they are not optimized to be executed by the new low-cost and high-efficiency microcontrollers [32]. These remarks are in line with recent review findings in agriculture [24] and reflect a problem already specified in the wider IoT area [4,33].

Trying to bridge this gap, the proposed solution indicates that, for water usage characterization/report delivery, a quite accurate model can now be trained, using flexible tools, be executed on the end device and communicate its classification reports using almost negligible power and bandwidth resources. Combining decentralized intelligence and low-cost design, provision is made for reduced to null amount of information to travel toward the cloud. These arrangements are addressing data privacy and reliability issues as well.

### 2.2. Functionality Overview and Component Selection

**Figure 1:**
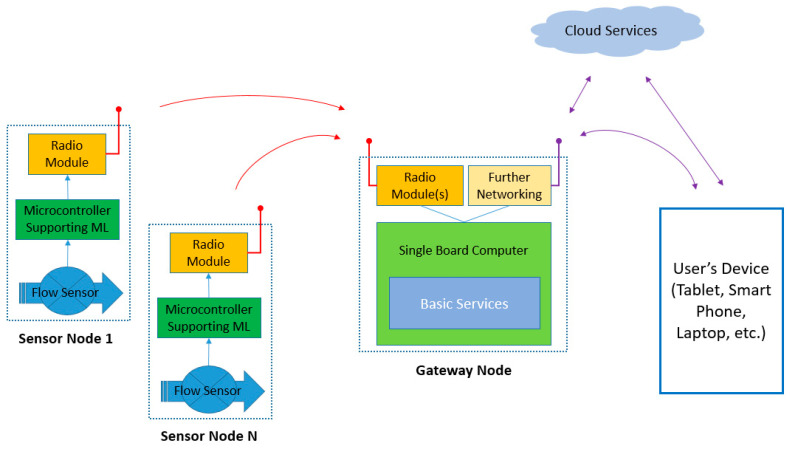
Functionality overview of the proposed water usage event characterization solution.

This section reports briefly on the components being selected as well as on their role, in order to develop a system capable of intercepting and characterizing water usage events. This system includes sensor nodes, placed in situ, at the edge points where the water is actually being used, as well as the suitable sink/gateway node(s) able to collect the reports delivered by the aforementioned peripheral nodes. The “key” point of the approach being presented is that the edge (sensor) nodes, apart from collecting time series corresponding to events containing the instantaneous water consumption data, are “smart” enough to classify these events into categories of proper or improper use of water, without assistance from external entities. Thus, via this “filtering”, only the classification reports have to travel toward the gateway and the cloud (if the latter is necessary). The analytical (low quality and high volume) information of the instantaneous water consumption might flood the network infrastructures and exhaust the batteries of the edge nodes. The user can easily monitor the operation of the whole system via their portable equipment (e.g., their tablet, smart phone, or laptop) using conventional connectivity options (e.g., Wi-Fi or 3G/4G), either locally or remotely (e.g., via a virtual private networking (VPN) service). The proposed architecture is depicted in Figure 1.

The proposed implementation exploited the experience gained during the activities described in [11] with the excellent Arduino Nano 33 BLE Sense [34] microcontroller that offers plenty of sensors and connectivity options, but utilized an even newer generation of cheaper microcontroller modules that were able to host and to execute composite machine learning algorithms, at the same price levels with the “traditional” units. For this reason, the Raspberry Pi Pico [35] microcontroller board (that costs about 6€) was selected, which, apart from its very attractive price, has fluent processing power and memory (due to its new RP2040 chip). More specifically, the Raspberry Pi Pico unit, grace at its new RP2040 chip, has fluent processing power and memory, that allows for larger and faster program execution compared to the typical Arduino Uno [36] standard, as it exhibits 64 times more flash memory (i.e., program memory), 128 times more random access memory (RAM) and a much faster dual-core processor. Consequently, the Raspberry Pi Pico board was able to support, apart from the basic water consumption metering process, the necessary machine learning functionality to invoke the corresponding water usage alert message generation. For the final deployment, the absence of a radio interface on the Raspberry Pi Pico unit was counterbalanced by the adoption of a cost effective microcontroller board, running at 8 MHz and equipped with a LoRa radio, namely a LoRa32u4 unit [37]. For programming both systems, the preferred option was the well-supported Arduino IDE [38] environment. During the implementation and testing stages, an ESP8266 based module [39], namely an ESP-01 unit, offering Wi-Fi connectivity, was utilized.

The water flow meter device is a Hall-effect counter sensor (YF-S201 [40] model), which can detect the flow changes as the water passes through it and the rotor rolls. Furthermore, the MIT App Inventor cloud-based programming environment [41] was selected for the easy creation of a mobile application for inspecting the water usage activity, via the smart phone/tablet device of the user.

To add machine learning functionality, it was necessary to prepare and incorporate a trained artificial neural network (ANN) model into the software running on the Raspberry Pi Pico. An artificial neural network is based on the operation of neurons in the human brain. This structure has one input layer, one or more hidden layers, being interconnected, and an output layer for delivering the results. A very simple and efficient manner to prepare (i.e., to train and to extract/compile) a suitable ANN model was the Edge Impulse [42] cloud environment. The latter processing environment incorporates the functionality of the TensorFlow Lite engine for training neural networks. More specifically, it is equipped with fluent graphical interface and network connectivity options for importing sensor data, designing the ANN model, applying assistive processing blocks, for creating, testing and deploying the final version of it. Finally, the coefficients describing the ANN are stored in the memory of the Raspberry Pi Pico microcontroller, and thus the AI algorithm can be executed on a device with comparatively low but enough capacity, in terms of processing power and RAM. The Edge Impulse platform, from February of 2022, provides full support from the Raspberry Pi Pico board.

The gateway node, gathers the classification decision information from the peripheral (edge) sensor nodes, stores and makes it available for the end device (e.g., smart phone, tablet or laptop) of the user, via common network services installed on it, or posts the information to the cloud, for better visualization and post-processing. Details referred to the latter choice are beyond the scope of this research work.

## 3. Implementation Details

**Figure 2:**
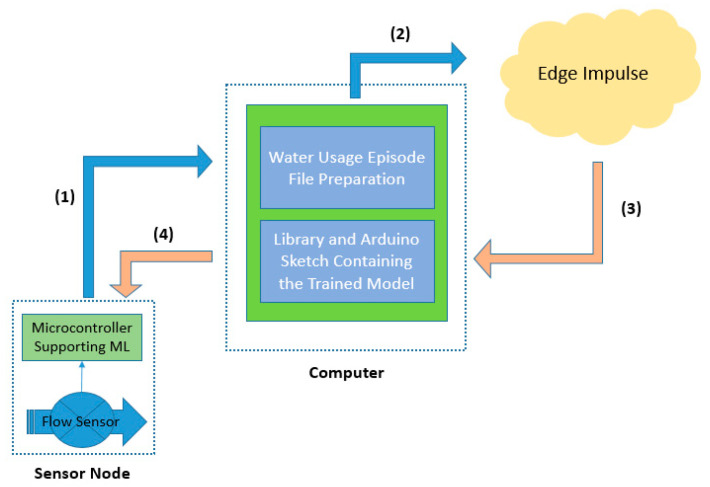
The analytic steps being necessary for the training of the proposed water usage event characterization solution.

In accordance with the design and functionality directions provided in Section 2.2, Section 3 is dedicated in presenting characteristic details of the implementation process. The analytic steps being followed for the training are illustrated in Figure 2.

More specifically, the basic water flow sensing unit connection and programming arrangements are highlighted, in order to gather efficient data for training the ANN model (step 1), and thus, to add machine learning capabilities to the whole system. The details for this training are also explained (steps 2 and 3), as well as the incorporation of the trained ANN model into the microcontroller of the flow-metering system (step 4) for enhancing its functionality. In parallel, the corresponding network node(s) arrangements are discussed, as well as the characteristics of a pairing end-user mobile application, for the delivery of a fluently working solution.

### 3.1. Initial Sensor Node Preparation

The Raspberry Pi Pico is a 3.3 V level unit, for this reason, the flow sensor was connected to its 3.3 V supply pin, in order to generate 3.3 V logic compatible pulse signals to its output. The 3.3 V level was adequate for the operation of the specific flow metering device being selected. Furthermore, the output of the latter sensor was connected with an interrupt (input) digital pin of the microcontroller, and the ground pins of both components were also wired together. The sensor was connected to a testing tap via a pipe, and thus, it could be exposed to a variety of water consumption scenarios potentially being invoked by human, according to empirical assumptions.

The Arduino IDE environment was customized properly by downloading and installing the necessary libraries corresponding to the Raspberry Pi Pico, according to the instructions of the its official page, for facilitating the programming process of the microcontroller, via a computer through a USB port connection.

The pulses that the flow sensor was generating correspond to the rotations of its blades and thus to the water flow passing through it. More specifically, according to the basic algorithm, as the flow sensor signal generated a pulse signal any time 2.22 mL water quantity, approximately, passed through it, the Raspberry Pi Pico intercepted these pulses as interrupt triggers to be counted and, in turn, calculated an one-second average value corresponding to the water flow (in mL). The sequence of these flow values was output to the serial port of the microcontroller. After compiling the program (sketch) and uploading it to the Raspberry Pi Pico board, the sequence of the flow measurements was acquired via the USB cable. The latter measurements were fed into the machine learning platform, in order to train the suitable ANN model, as the Edge Impulse environment offers options for automated uploading of the values being measured.

### 3.2. Training the Neural Network

The corresponding ANN model to be generated had to be simple and lightweight enough for the microcontroller’s potential but still precise enough. In this regard, the system was trained to recognize three characteristic kinds of water utilization profiles: the Normal Use or NU, Water Leak or WL and Water Waste or WW. The proper training of an ANN requires data series corresponding to each of these categories to be collected and to be uploaded to the Edge Impulse engine. The total data length was 5 h 55 min 47 s (148 files) for all three cases. According to Edge Impulse platform requirements, the duration of the data length had to be approximately the same for all categories, in order for the final model to be more accurate. Nevertheless, the number of profiles for each case may differ (NU: 69, WL: 44, WW: 44 profiles).

**Figure 3:**
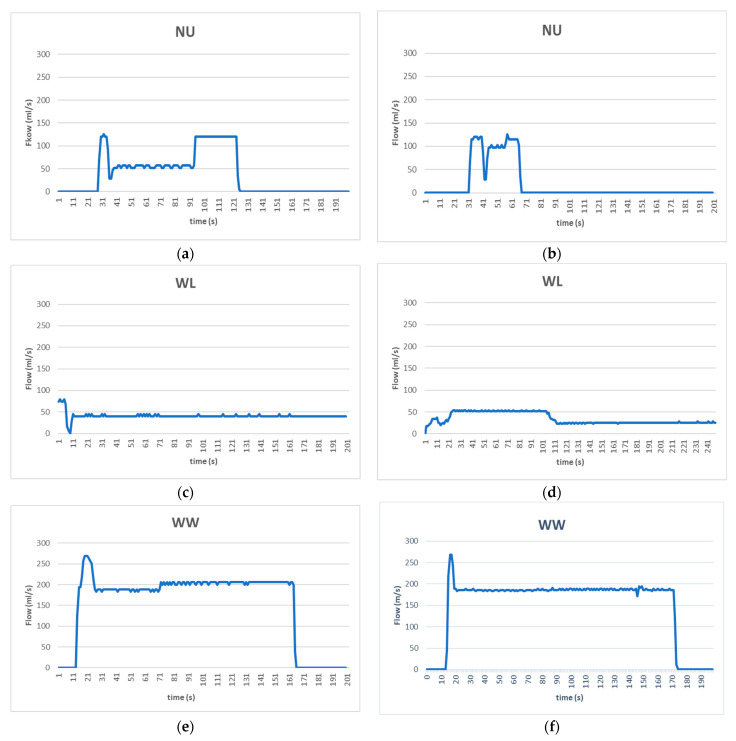
(**a**,**b**) Normal Use profiles; (**c**,**d**) Water Leak profiles; (**e**,**f**) Water Waste profiles.

During the profile collection process, the lowest flow value that the flow sensor could record was about 10–15 mL/s, while the maximum flow being recorded was in the range between 250 and 280 mL/s. The network was trained using empirical data based on human observations for classifying samples (water usage episodes) into categories. In general, NU profiles were created so as to contain low to moderate flow values and having duration below 180 s, making the training pattern hypothesis that a non-WL water usage scenario would last for 3 min at maximum. Similarly, it was assumed that WL profiles exhibited continuous flow duration of more than 180 s and that most WW profiles had flow consumption over 160 mL/s and duration of more than 160 s, as it would be more likable for the classification experiment, during the episodes to use water for shorter time and at lower flow rate. Some typical profiles for each category are given in Figure 3, where the water flow was measured in ml/s and the time was measured in seconds (s). For each category, there is a diversification among the profiles being recorded and fed to the training system. This diversification results in increased accuracy under real-world conditions.

In the next stage, the water flow data (raw data) were uploaded to the Edge Impulse cloud platform, via the Data Acquisition menu category, and were split into training and testing data, automatically, while the data labelling was performed manually.

For training of the ANN model, the window size was set at 200,000 ms (i.e., 200 s), according to the profiles that were fed into the training system and by taking into consideration the maximum time that a person might use the tap. Similarly, the window increase was set at 1000 ms (i.e., at 1 s) and the frequency at 1 Hz (i.e., for 1 sps sampling rate). Furthermore, “Raw Data” was selected as the preferred processing block and “Classification (Keras)” as the ANN learning block. The option “Raw Data” means that no additional prepossessing was made (e.g., a spectral characteristics extraction) before using the original data for the training process. This option does not reduce the number of features to be fed to the input layer of the network, but also preserves as many characteristics of the initial data as possible and, as it is explained right below, it fits easily in the microcontroller being selected. Furthermore, the number of training cycles was set to the moderate value of 50, to avoid overfitting, and the learning rate at 0.0005, via the NN Classifier configuration section, as the Edge Impulse suggests. The final neural network structure has an input layer with 200 features (window size), two hidden layers, with the first one to have 20 neurons and the second one 10 neurons, and an output layer with 3 classes (NU, WL, WW). This architecture for the NN provided an optimal combination between performance and computer resource allocation (i.e., model accuracy versus time needed for a decision to be made and memory size needed for hosting the program in the flash and for executing it in RAM). For the specific model, in the quantized version, the RAM usage was 1.9 KB and the flash memory usage was 22.5 KB, values that are far below the capacity limit of the Raspberry Pi Pico unit. It must be noted though that during the actual operation of the microcontroller, more memory will be needed as along with the NN model coexist several variables and code parts dedicated to other tasks.

The Edge Impulse platform allows for easy experimentation with various candidate settings and for saving the model with the best performance after the end of the training process. Finally, there is the option to download the model from the Edge Impulse cloud platform, via the “Deployment” section of Edge Impulse menu category, as code that includes library and sketches to be compiled and uploaded to the microcontroller via the Arduino IDE environment.

### 3.3. Sensor Node Software Enhancement

As explained in Section 3.2, the code generated by the Edge Impulse platform, in the form of a generic Arduino library, provides customizable examples (sketches) for the Arduino environment, with the Raspberry Pi Pico board to be among the models being supported, and thus, being compatible with the generated model parameters. The selection of the “Arduino library” option (instead of the tailored firmware output one) provides freedom to combine the machine learning engine with further algorithmic behaviors being necessary to be executed by the hosting microcontroller.

In this regard, the final software running on the microcontroller had to be updated so as to be able to perform (almost simultaneously) some simple but sharp calculations/tasks of different time granularity:

- Intercept the interrupt signals corresponding to the rotor roll pulses of the water flow sensor module;
- Calculate the instantaneous water consumption, at a fixed and specific rate, typically 1 or 2 times per second, update the aggregate metrics, and trigger the classification process every time the predefined number of samples (i.e., 200) was gathered;
- Deliver system status data and water usage reports via USB to the hosting computer, or wirelessly to a gateway node or to the operator’s smart phone/tablet;

As expected, the above tasks had to be performed without blocking or delaying each other, constraints that required meticulous programming (e.g., using timer events) to achieve fluent operation. Optimally, the delivery of information toward the gateway had to take place once, after the end of each classification process utilizing the 200 consecutive samples. Nevertheless, for debugging or training purposes, all 200 values had to be transmitted toward the gateway node. Communication with the LoRa32u4 radio module was achieved through the serial TTL level port of the microcontroller.

### 3.4. Gateway Node and User-End Software

For the reception (and the inspection) of the remote alerts through Wi-Fi, an android smart phone or a tablet device, which most modern people are familiar with, was a satisfactory solution. The MIT App Inventor environment was utilized in order to deploy a simple monitoring application. The necessary programming was completed using visual blocks, based on the information provided in [43,44].

The initial deployment involved direct connection between the smart water sensor node and the end user equipment (e.g., a tablet device), typically through a Wi-Fi connection link. This solution is not optimal if multiple sensors units exist and deliver water usage reports in parallel. Furthermore, the latter sensors may be placed at comparatively long distances from the user. These facts made necessary the development of a gateway/sink node to gather the corresponding data and the migration to LoRa radio links.

For implementing the latter gateway node, a Raspberry Pi 3 Model A+ had been selected [45], due to its reduced size and energy footprint and its fluent programming and interfacing options. The Raspberry Pi Model 3 A+ unit allows for fast implementation of code that intercepts the data reports from the peripheral smart sensor nodes, storing them into files or a simple database, and making them available via the proper TCP/IP based service. This request could be either asynchronous or periodic (i.e., generated by a proper application running on the user’s mobile phone). These tasks are served using python and Linux shell scripts, inter process communication (IPC) techniques exploiting IP sockets, and the activation of preexisting applications such as the Apache web server, the SSH server and/or a Virtual Private Networking (VPN) service. Furthermore, the gateway node, properly combined with VPN networking techniques, assured monitoring functions from distant locations, based on the availability of Wide Area Network (WAN) wired or wireless technologies (i.e., 3G/4G, DSL, etc.).

### 3.5. Summary of IoT Deployment Steps

The Edge AI tasks had to be performed fluently, while deployment in open-field environments using long-range radios, such as LoRa, was an important priority. The final functionality being implemented can be summarized in the following steps/cases:

1. Use a Wi-Fi radio transceiver (e.g., an ESP-01 module), attached to the sensor node, to provide communication between the sensor node and the user’s smart phone/tablet, for testing purposes, during the initial deployment;
2. Use a Raspberry Pi Model 3 A+ and a LoRa radio module as a LoRa gateway/web server, in conjunction with the LoRa radio transceiver modules being attached to the (preferably more than one) smart sensor nodes;
3. Increase user-friendliness by adding services using the Raspberry Pi Model 3 A+ unit of the gateway node and well-known web-based applications.

Case 1 was suitable for verifying the basic wireless connectivity potential of the sensor node via the tablet/smart phone device of the user, being nearby the sensor. This arrangement made easy for the user to inspect the status of the water activity characterization system for one smart sensor and from short distances.

The need to have a more complete on-demand view of the status of more than one water use points, at increased distance, was favoring the adoption of a local gateway node facilitating the whole monitoring process, as explained in case 2. The sensor nodes were sending water usage notifications toward this local gateway, over LoRa. It must be noted though that the TCP/IP technology, as a solution for the delivery of data (i.e., the sporadic metadata) from the sensors to the gateway, is not optimal, in terms of energy consumption, complexity and range coverage. Indeed, in a typical application scenario, the distance between the sensor nodes and the gateway node is limited to a hundred meters, approximately. If willing to extend this distance to the kilometer range or beyond, without special and expensive equipment, transceivers utilizing technologies such as LoRa are more suitable.

In case of the LoRa solution, the LoRa32u4 board, as a transceiver, was the optimal selection for both the sensor and gateway nodes, due to its low cost and its easy programming. The RadioHead software package [46] is a very efficient library that supports several critical LoRa protocol functions, and thus, it was adopted for adjusting the LoRa32u4 modules. These modules were programmed easily via the Arduino IDE environment. Consequently, the microcontroller of each sensor node was connected (typically via its hardware serial TTL interface) with a LoRa32u4 board in order to relay the water usage information from the machine learning engine toward the gateway node. A Lora32u4 board was also connected via USB with the Raspberry Pi 3 Model A+ unit implementing the gateway functions. The necessary code was written in python to bridge the serial port of the LoRa32u4 board with an IP socket service running on the gateway node.

**Figure 4:**
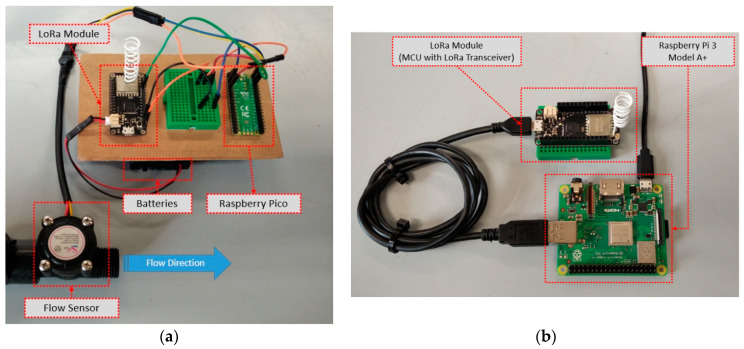
(**a**) Smart water sensor node deployment using Raspberry Pi Pico and LoRa radio; (**b**) Gateway/sink node implementation using Raspberry Pi 3 Model A+ and LoRa radio.

Characteristic deployment arrangements are depicted in Figure 4a,b. More specifically, Figure 4a depicts the smart water sensor node implementation using a Raspberry Pi Pico unit and a LoRa radio, while in Figure 4b the gateway/sink node implementation is depicted using a Raspberry Pi 3 Model A+ and a LoRa radio. The information exchanged between the LoRa radios was packetized and encrypted using the RadioHead library and the Arduino Cryptography Library [47], in order to hide the sensitive data from non-authorized users.

Initial experiments were performed using USB powering via the hosting computer and/or power banks. Later updates included LiPo or Li-ion batteries, mainly of 18650 type which are cheap and robust, as well as small photovoltaic panels (e.g., 2 W units). It must be noted though that the absence of a permanent power supply source nearby is not always the rule, and thus the operation of the alerting system was facilitated.

## 4. Results and Evaluation

This work is putting emphasis on intercepting water usage events and on characterizing them properly. Via fluently-working machine learning techniques, applied at the edges of the network, the amount of information that needs to travel from the peripheral nodes to the central node and the cloud is minimized. This fact signifies reduced communication load and energy consumption, and better autonomy and privacy. The adoption of simple, long-range and low-energy radios facilitates the whole process. Relevant details are given into the following Section 4.1, Section 4.2, Section 4.3 and Section 4.4.

### 4.1. Testing the Acuracy of the Model

For classification evaluation algorithms, accuracy is the most frequently used indicator, and it is defined as the proportion of the correctly classified samples to the total number of samples. After the training process, based on the testing data, the system generated the right outcome for the NU category with 77.8% accuracy. Similarly, for the WW and WL categories, 100% success was achieved, according to Edge Impulse cloud environment. These performance results made the final model to have a 98.5% expected accuracy, using the testing data set, in the Quantized (int8) version.

**Figure 5:**
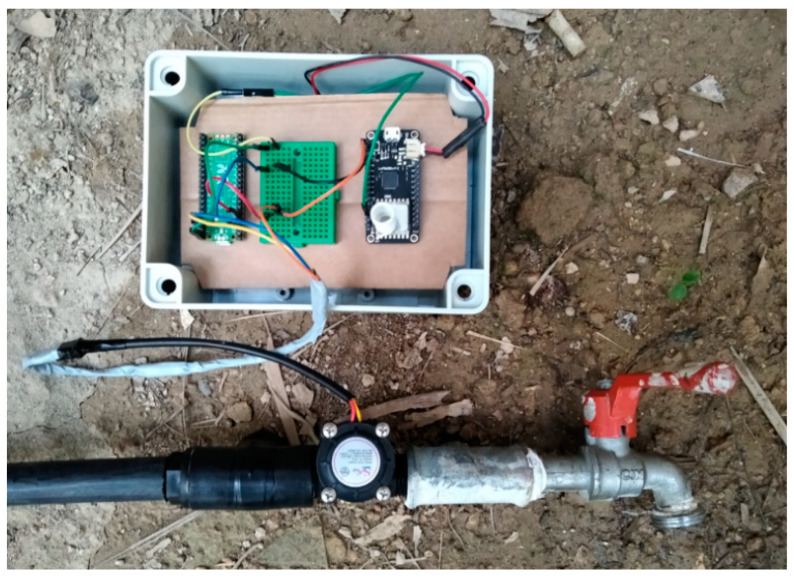
The proposed sensor node connected in-line with a water tap.

At next stage, actual water consumption episodes of known type (i.e., NU, WW or WL) had to be invoked, by rotating the tap head properly, thus letting the proposed machine learning engine to perform classification according to the flow data being collected (i.e., in chunks of 200 consecutive values). The corresponding results were recorded. Figure 5 depicts the proposed sensor node connected in-line with a water tap. This process was matching the steps being followed during the training stage of the system.

**Figure 6:**
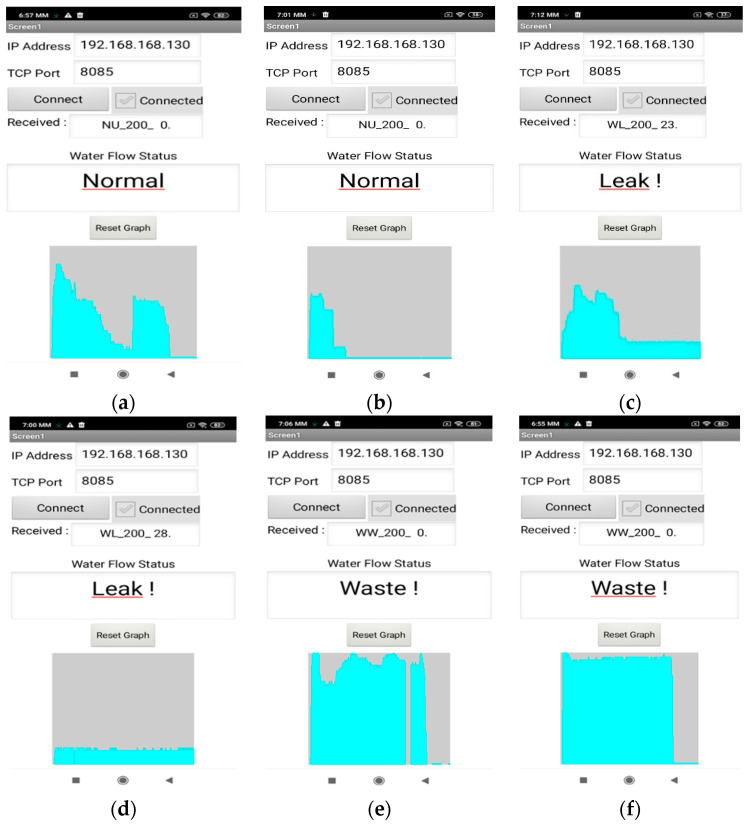
(**a**–**f**) Indicative smart phone screenshots during the in situ testing process, reflecting typical water usage characterization decisions for the categories NU, WL and WW, respectively.

It must be noted that the in-parallel visual inspection of the ongoing process was drastically facilitating the experiments. More specifically, further arrangements were made in order for the whole sequence of the analytical flow readings to arrive to the smart phone/tablet device, using a modified version of the application created for the end user (as presented in Section 3.4). This application variant provided detailed real-time graphs (into the form of histograms) reflecting the instantaneous water consumption during each episode, for direct comparison and adjustments. Figure 6a–f illustrate indicative smart phone screenshots reflecting typical water usage characterization decisions during the actual testing process, corresponding to the NU, WL and WW categories, respectively.

The combination of the trained ANN model implementation with simple more conventional programming techniques was improving the accuracy and the response times of the system being presented. For instance, the in situ module logic was modified so as to ignore the zero-flow events, as an episode (i.e., event) started being recorded only after the arrival of the first non-zero flow value.

Table 1 contains the confusion matrix that corresponds to the testing of the real system, after classifying 100 water consumption episodes. The processing of the data being collected revealed that the actual accuracy was 91% (i.e., 91 over 100 samples were classified correctly), after testing the model with user-generated water consumption profiles, using the proposed smart flow metering system. It is important to mention that the model could clearly recognise the undesirable WL profiles, achieving accuracy values reaching 100%. On the other hand, there were some incorrect predictions, where the neural model was classifying an actual WW scenario as NU or WL (with percentages 5.1% and 7.7%, respectively). In fewer cases, the model was classifying an NU as WW or WL (with percentages equal to 2.8%). These failures can be attributed to the fact that there was a small area where the borders of those categories were overlapped, thus confusing the neural network classifier. An additional 0.4 certainty threshold was programmed on the microcontroller for more reliable characterizations. This performance is close to the one expected according to the testing of the model. The overall performance is lower than the one achieved by other machine learning approaches [23] using more composite systems, but remains high and can be easily achieved by the proposed low-cost equipment. The accuracy can be further improved by using more extensive training and samples.

### 4.2. Networking and Power Consumption Issues

According to the specifications of the experimental system being presented, although 200 consecutive samples had to be recorded before a classification decision to be make, this decision was taken locally, and thus only the (final) characterization message had to travel toward the gateway (and to the end user) instead of 200 messages containing the corresponding analytical flow values. The packet payload information needed to travel from the peripheral sensor nodes toward the gateway node did not exceed 10 bytes in binary format, thus resulting in a bellow 50-byte description per episode in textual format, in the final log files on the Raspberry Pi Model 3 A+ unit of the gateway. The size requirements of the analytical data would be roughly 200 times higher. In addition to that, the cost for performing the classification at the central node was not necessary any more.

**Figure 7:**
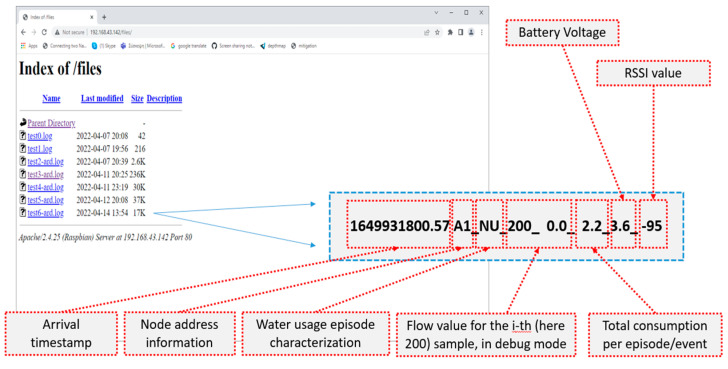
Characteristic details of the water flow episode/event specific information as stored into the log files on the Raspberry Pi Model 3 A+ unit implementing the gateway node.

Figure 7 provides indicative details of the water flow episode/event specific information as stored into the log files on the Raspberry Pi Model 3 A+ unit implementing the gateway node functionality. These files were directly available through the Apache web server and typically contained an arrival timestamp, node address, episode type (i.e., NU/WW/WL), flow value per each sample into a specific episode (in debug mode only), total water consumption per episode, as well as sensor battery voltage and RSSI indicator.

Some stability problems were experienced when using the highest baud rate (i.e., the 115,200 bps value) between the Raspberry Pi Pico and the LoRa32u4 module. For this reason the data rate was set to the “safe” 38,400 bps value.

**Figure 8:**
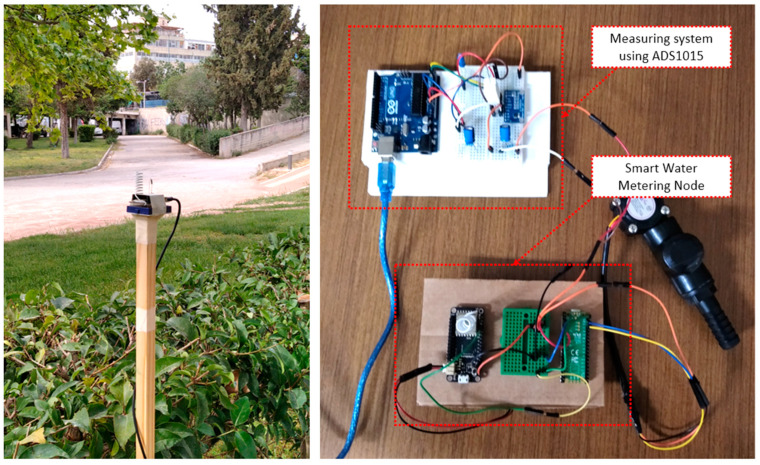
Experiments for testing the range coverage (**left**) and the energy consumption (**right**) of the prototype sensor nodes.

The techniques being followed for testing the effective communication range of the proposed system were utilizing the methods presented in [7,48]. The gateway node, apart from the water flow specific information, for each node, was collecting assistive data, such as sensor battery status and received signal strength indicator (RSSI). The latter information was collected for sensor nodes being at various distances from the gateway node, for both Wi-Fi and LoRa radio cases. The left part of Figure 8 depicts a LoRa radio transceiver during the in situ radio coverage experiments. According to results being gathered, by using ESP-01 Wi-Fi transceivers, the maximum range coverage was at about 100 m, while by using LoRa modules with custom wire antennas the communication distance was extended to 300 m in free space. By using standard but still cheap antennas, the LoRa link scenario was easily achieving communication coverage of above 1 km. These results are justified by the fact that the receiver sensitivity limit for nodes equipped with Wi-Fi radios was around −90 dBm, while for the LoRa, the sensitivity being achieved was reaching the −130 dBm level.

In order to better capture and study the short-scale dynamics of the smart sensor modes, a measuring circuit was built, according to the directions provided in [49]. More specifically, an Arduino Uno board was utilized to calculate the voltage drops over a resistor of known value, connected in series with the load of interest (i.e., the smart water sensor node); the right part of Figure 8 depicts the corresponding experimental setup. The actual measuring process was performed via a separate ADC module (namely an ADS1015 unit) capable of true differential measurements, of satisfactory resolution (i.e., of 12 bits) and of adjustable gain. The communication of this module with the hosting Arduino board was completed using an I2C interface. The presence of the Arduino Uno unit allowed for the additional processing of data and quick graphical inspection. Consequently, for the system under testing, amperage consumption traces could be easily captured, at a typical time resolution of 100 sps and at an approximate amperage resolution of 1 mA, via the Serial Monitor or the Serial Plotter component of the Arduino IDE environment. By using the specific measuring setup, several results were collected. The behavior of the sensor nodes was on the focus of this study, as, typically, the gateway node was considered of having fixed power supply and its consumption was around 250 mA.

More specifically, the consumption of a bare node, equipped only with a Raspberry Pi Pico unit was 27 mA, approximately, with the water flow metering unit to absorb 3–4 mA of this quantity. When activating the radio modules on the system and letting them transmit information, further data were collected. For debugging purposes, apart from the standard settings where only the water usage decision was reported, the analytical flow data could also be transmitted toward the gateway, limited only by the maximum data rate being supported by the selected radio modules.

**Figure 9:**
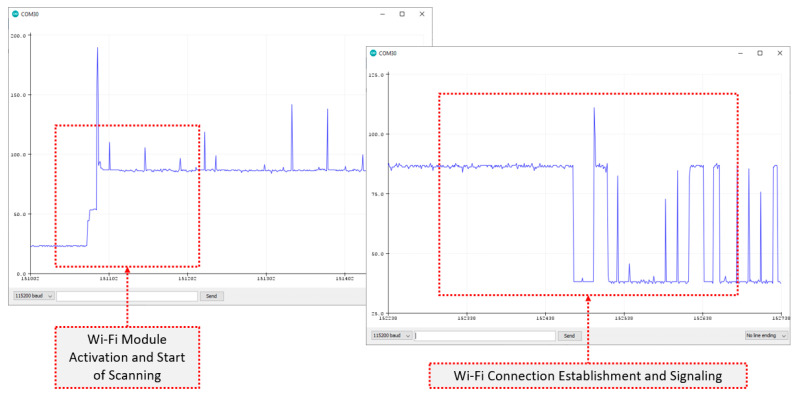
Short time dynamics of the mandatory scanning and connection establishment stages, following the activation of the Wi-Fi radio module that smart sensors were equipped with.

Referring to the Wi-Fi communication case, Figure 9 provides characteristic details of the short time dynamics of the scanning and connection establishment stages that were mandatory before the utilization of the radio modules. The inspection of the results revealed that the scanning process was extremely energy-consuming, reaching the level of 90 mA (in total) with additional and non-negligible sporadic spikes exceeding that level. The whole scanning process lasted for 2 to 3 s, and after that, the overall consumption was stabilized to the 40 mA level, with peaks of additional 50 mA corresponding to the water flow event reports toward the gateway. The high cost for the Wi-Fi initialization link (especially in optimized radio sleep/wakeup scenarios), along with its limited range coverage were favoring the assessment of other communication alternatives, such as LoRa.

**Figure 10:**
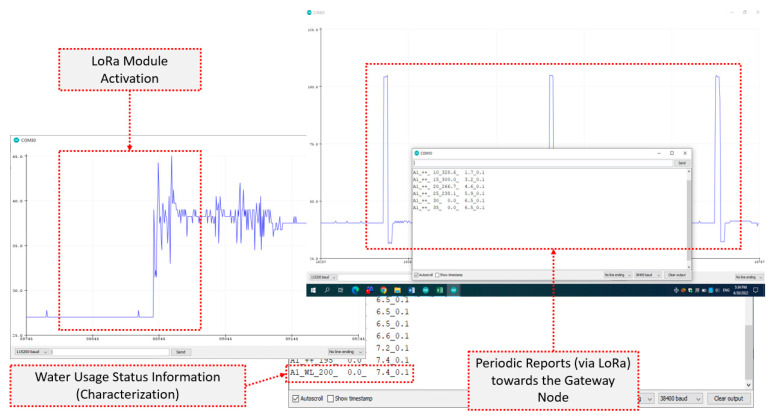
Characteristic short-time dynamics for the LoRa communication alternative: From the LoRa module activation (**left**) to the energy peaks reflecting the packet transmission events (**top right**) and to the corresponding textual information content as intercepted by the gateway (**bottom right**).

Similarly, Figure 10 depicts characteristic short-time dynamics for the LoRa communication alternative. Namely, from the LoRa module activation (left) to the energy peaks reflecting the water usage notification packet transmission events (top right) and to the corresponding textual information content as intercepted by the gateway (bottom right). The LoRa32u4 LoRa board consumed 12–13 mA, approximately, at idling, with the radio enabled, while the transmission events at the standard radio parameter settings (i.e., having Coding Rate—CR set to 4/5, Bandwidth—BW to 128 kHz, Spreading Factor—SF set to 7) and with the transmit power at 15 dBm, resulted in spikes of 70 mA (at 3.3 V), having an approximate duration of 50 ms, thus requiring around 12 mJ each. It must be noted that the whole process lacked the high connection establishment cost (in both time and energy) characterizing the Wi-Fi case. The tradeoff of LoRa was the far lower communication rate, which was not an issue for the specific application case that only a few bytes had to be transmitted per sensor unit, every 2 to 3 min, at the fastest utilization activity scenario.

According to the overall performance description presented herein, it can be inferred that typically, the benefits of the pilot implementation being discussed were maximized in application cases where many water consumption check points were needed, spread into an area of a few kilometres.

### 4.3. Node Cost Issues

The total cost of each of the discussed nodes, after adding the 6€ for the Raspberry Pi Pico unit, the 15€ for the LoRa equipped module, the 8€ for the YF-S201 flow sensor, the 8€ for LiPo batteries and the 5€ needed for a good-quality plastic enclosure box, was around 42€. The utilization of a LoRa transceiver instead of a typical Wi-Fi radio saved energy and offered improved distance coverage. The decision of using the LoRa32u4 board added some extra cost (of about 5€, compared with a bare LoRa chip) but provided further GPIO pins and connectivity options, as well as fast programming and testing of the diverse communication and arithmetic processing variants, thus counterbalancing the almost 15 min of time required for the compilation of the code containing the trained neural network model destined for the Raspberry Pi Pico unit. The gateway node needed 30€ for a Raspberry Pi Model 3 A+, 15€ for the LoRa32u4 board, 5€ for a plastic enclosure box, and 5€ for a power supply, resulting in cost below 60€.

### 4.4. Further Discussion

This work presented a pilot implementation targeted at intercepting water usage events and characterizing them properly, with the emphasis to be put on misuse cases, such as leakages or wastes. The rapid growth of electronics and of the pairing software allowed for very cost-effective but efficient solutions, with cutting-edge features. Indeed, the adoption of machine learning techniques at the edge points (i.e., where the water sensors are) was drastically reducing the amount of information that needed to travel from the peripheral nodes to the central node and the cloud. This fact resulted in reduced communication load and energy consumption, while it increased autonomy and privacy. The focus was put on the in situ processing and the pairing with simple, long-range and low-energy radios, e.g., the LoRa technology ones. The water usage episodes the experimental system was trained to intercept were of comparatively short duration, but the software and hardware methods being used, and the accuracy being achieved, make the proposed arrangements, only with minor configuration modifications, to be applicable for supporting a wide variety of water preservation/misuse detection scenarios. Apparently, several issues are still open, requiring more elaboration for the delivery of an out-of-the-box solution.

The time interval between the fixed, in number (e.g., 200), consecutive flow data required for a characterization decision, was set to 1 s during the training. The same trained model, can still be valid considering intervals of much longer value (e.g., of 30 s instead of 1 s), provided that the proper normalization in flow values will be made and that the activity will be classified in following the same pattern. Nevertheless, gathering richer data sets, reflecting further realistic use cases, can train the model more accurately, and is an apparent priority for wider applicability. This training can follow the same generic principles and methods described herein.

The option of using a bare LoRa chip with the Raspberry Pi Pico unit is amongst the future priorities toward a more commercially-friendly version of the prototype presented herein. While the adoption of the LoRa protocol allows for better flexibility, the LoRaWAN solution is also feasible, either via implementing the necessary protocol stack, via software on the 32u4 LoRa board, or by utilizing native LoRaWAN chips. Furthermore, these processes can become more efficient by introducing a sleep/wakeup energy management schema which will allow the Raspberry Pi Pico to wake up (via interrupts) whenever water flow activity is intercepted by the flow sensor. The task of the efficient powering the system at the absence of permanent power supply nearby is also quite challenging. Indeed, more than one alternative can be adopted, from using solar panels or a tiny wind generator, to pairing the rotating blades of the flow sensor unit with a tiny electric generator [50]. Finally, as the adoption of a Raspberry Pi Model 3 A+ as a central/gateway node was providing an adequate but poor level of functionality, via elementary web and archiving or database services, linking with well-known and more user-friendly cloud services is also a case worth investigating in the future.

## 5. Conclusions

In this paper, the synergy between several innovative and low-cost electronic components and software was exploited, in order to monitor and remotely report characteristic water consumption/misuse events. The whole approach introduces modern Edge AI techniques (i.e., combining IoT, ML and Edge Computing principles) that up until recently was not possible to be executed with traditional low-cost microcontrollers. The challenges for the delivery of a generally applicable and inexpensive alerting system for either urban or rural water resource usage were further highlighted. The system being presented can work in a decentralized manner as the amount of information that has to travel from the edges to the cloud is drastically reduced, or becomes practically unnecessary, thus resulting in energy requirement minimization and increased privacy. Only the final decision (water usage characterization) information has to be transmitted to the final user (e.g., the farmer), and the cloud is necessary only in case that the latter user is not nearby or asks for sophisticated information post processing.

As for the future, more optimized variants of the proposed system will be assessed, in terms of hardware selection (e.g., of flow sensor units), neural network model accuracy, networking options and energy autonomy. Great companies, such as Arduino, Raspberry, ESP or Adafruit, during their noble competition, will continue to produce excellent parts with leveraged application support potential. Finally, an out-of-the box version of the functionality being presented, of commercial standards, exploiting additional well-known services, and thus exhibiting increased user-friendliness, will be a significant future priority.

## Figures and Tables

**Table 1:** The confusion matrix corresponding to the trained neural network model, created by classifying 100 water consumption episodes, of specific (and known) type each.

Class	NU	WL	WW	Unknown
NU	91.7%	2.8%	2.8%	2.8%
WL	0.0%	100.0%	0.0%	0.0%
WW	5.1%	7.7%	84.6%	2.6%

## Data Availability

Available upon request.
